# Errata

**DOI:** 10.36660/abc.20230178

**Published:** 2023-04-27

Arq Bras Cardiol. 2023; 120(2):e20220247

No Artigo Original “Consumo de Oxigênio e Aptidão Cardiorrespiratória | Diferença entre Idade Cronológica e Biológica Estatísticas Cardiovasculares do Programa Boas Práticas em Cardiologia – Dados de um Hospital Público Terciário Brasileiro”, com número de DOI: https://doi.org/10.36660/abc.20220247, publicado no periódico Arquivos Brasileiros de Cardiologia, Arq Bras Cardiol. 2023; 120(2)e20220247, na página 1, corrigir o nome da autora Carolina Teixeira Cunha Érika para: Carolina Teixeira Cunha.

Na página 11 corrigir a referência 18 para: Passaglia LG, Brant LCC, Silva JLP, Nascimento BR, Ribeiro ALP. Text Messages to Promote Secondary Prevention after Acute Coronary Syndrome (IMPACS trial). Int J Cardiovasc Sci. 2021;35(2):202-13. doi:10.36660/ijcs.20200378.

Arq Bras Cardiol. 2023; 120(3):e20220649

No Ponto de Vista “As “Cinco Ondas Malignas” da Eletrocardiografia”, com número de DOI: https://doi.org/10.36660/abc.20220649, publicado no periódico Arquivos Brasileiros de Cardiologia, Arq Bras Cardiol. 2023; 120(3):e20220649, na página 1, corrigir o título do artigo em inglês para: The “Five Malignant Waves” of the Electrocardiography.

