# Supplementary material for: Errata
Source: Arq Bras Cardiol. 2023 Apr 27;120(5):e20230178. [Article in Portuguese] doi: 10.36660/abc.20230178 (PMC10263390; doi:10.36660/abc.20230178)
Supplement: Translation

# Translation: Erratum

Arq Bras Cardiol. 2023; 120(2):e20220247

In the Original Article “Cardiovascular Statistics from the Good Practices in Cardiology Program – Data from a Brazilian Tertiary Public Hospital”, with DOI: https://doi.org/10.36660/abc.20220247, published in the journal Arquivos Brasileiros de Cardiologia, Arq Bras Cardiol. 2023; 120(2)e20220247, on page 1, correct the author’s name: Carolina Teixeira Cunha Érika for: Carolina Teixeira Cunha.

On page 10 correct reference 18 to: Passaglia LG, Brant LCC, Silva JLP, Nascimento BR, Ribeiro ALP. Text Messages to Promote Secondary Prevention after Acute Coronary Syndrome (IMPACS trial). Int J Cardiovasc Sci. 2021;35(2):202-13. doi:10.36660/ijcs.20200378.

Arq Bras Cardiol. 2023; 120(3):e20220649

In the Viewpoint “The “Five Malignant Waves” of the Eletrocardiography”, with DOI: https://doi.org/10.36660/abc.20220649, published in the journal Arquivos Brasileiros de Cardiologia, Arq Bras Cardiol. 2023; 120(3):e20220649, on page 1, correct the title of the article in English to: The “Five Malignant Waves” of the Electrocardiography.
